# Efficacy of a church-based lifestyle intervention programme to control high normal blood pressure and/or high normal blood glucose in church members: a randomized controlled trial in Pretoria, South Africa

**DOI:** 10.1186/1471-2458-14-568

**Published:** 2014-06-06

**Authors:** Supa Pengpid, Karl Peltzer, Linda Skaal

**Affiliations:** 1 Department of Research Development & Innovation, University of Limpopo, Sovenga 0727, Turfloop, South Africa; 2 ASEAN Institute for Health Development, Mahidol University, Salaya 73170, Phutthamonthon, Nakhonpathom, Thailand; 3 Department of Psychology, University of Limpopo, Sovenga 0727, Turfloop, South Africa; 4 HIV, AIDS, TB, and STIs (HAST), Human Sciences Research Council (HSRC), Pretoria 0001, South Africa; 5 Department of Public Health, University of Limpopo, Sovenga 0727, Turfloop, South Africa

## Abstract

**Background:**

In persons 15 years and above in South Africa the prevalence of pre-diabetes and diabetes has been estimated at 9.1% and 9.6%, respectively, and the prevalence of systolic prehypertension and hypertension, 38.2% and 24.6%, respectively. Elevated blood glucose and elevated blood pressure are prototype of preventable chronic cardiovascular disease risk factors.

Lifestyle interventions have been shown to control high normal blood pressure and/or high normal blood glucose.

**Methods/Design:**

This study proposes to evaluate the efficacy of a community (church)-based lifestyle intervention programme to control high normal blood pressure and/or high normal blood glucose in church members in a randomized controlled trial in Gauteng, South Africa. The objectives are to: (1) measure non-communicable diseases profile, including hypertension and diabetes, health behaviours, weight management and psychological distress of church members; (2) measure the reduction of blood glucose and blood pressure levels after the intervention; (3) prevent the development of impaired glucose tolerance; (4) compare health behaviours, weight management and psychological distress, blood glucose and blood pressure levels between intervention and control groups, and within group during 6, 12, 24 and 36 months during and post intervention. The study will use a group-randomized design, recruiting 300 church members from 12 churches. Churches will be randomly assigned to experimental and control conditions.

**Discussion:**

Lifestyle interventions may prevent from the development of high blood pressure and/or diabetes. The findings will impact public health and will enable the health ministry to formulate policy related to lifestyle interventions to control blood pressure and glucose.

**Trial registration number:**

PACTR201105000297151

## Background

In the first South African National Health and Nutrition Examination Survey (SANHANES-1) [1] of 2012, a prevalence of 9.1% and 9.6% of prediabetes and diabetes in persons 15 years and above, respectively was found. In the age groups 35–44, 45–54, 55–64, and 65 years and more, the prevalence of prediabetes increased from 9.0%, 11.2%, 13.9% to 19.9%, while diabetes was 4.3%, 16.7%, 24.4% and 19.0%, respectively. In the same SANHANES-1 [1], a prevalence of 38.2% and 20.0% systolic and diastolic prehypertensive blood pressure, respectively, and 24.6% and 9.1% systolic and diastolic hypertension, respectively, in persons 15 years and above was found. In the age groups 35–44, 45–54, 55–64, and 65 years and more, the prevalence of systolic prehypertension was 45.2%, 37.1%, 31.7% and 24.3%, respectively, and the prevalence of systolic hypertension was 21.9%, 39.2%, 50.5% and 63.7%, respectively. Elevated blood glucose and elevated blood pressure are prototype of preventable chronic cardiovascular disease risk factors [2].

Lifestyle interventions have been shown to ameliorate or prevent the progression of prediabetes and prehypertension to diabetes and hypertension [3,4]. Such a non-pharmacological approach to primary prevention and disease interruption carries enormous public health significance [3]. Improving diet and physical activity can prevent type 2 diabetes among those at high risk in a very short time [5]. In trials conducted in China, Finland and the USA, for example, study participants have significantly improved their diet and/or physical activity, and shown improved levels of blood pressure, blood glucose, cholesterol and triglycerides within one year of commencing a programme, with improvements continuing for at least six years. The incidence of diabetes was reduced by almost 60% in both Finland and the USA, and by over 30% in China [6,7]. Orozco et al. [8] conclude that interventions aimed at increasing exercise combined with diet are able to decrease the incidence of type 2 diabetes in high risk groups (people with impaired glucose tolerance or the metabolic syndrome). Likewise, lifestyle modifications can reduce blood pressure and lower cardiovascular risk. Several clinical trials investigated the efficacy of non-pharmacological interventions and lifestyle modifications to reduce BP. Best evidence from randomized controlled trials supports BP-lowering effects of weight loss, the Dietary Approaches to Stop Hypertension (DASH) diet, and dietary sodium (Na+) reduction in those with prehypertension [9]. Established recommendations include weight loss, sodium reduction, and increased physical activity. PREMIER studied the effects of lifestyle interventions based on established recommendations alone and with the addition of the Dietary Approaches to Stop Hypertension (DASH) dietary pattern [10]. Participants with prehypertension or stage-1 hypertension were randomly assigned to an advice only control group, a 6-month intensive behavioural intervention group of established recommendations (EST), or an established recommendations plus DASH group (EST + DASH). Both EST and EST + DASH reduced the primary outcome variable, systolic blood pressure [10]. Maruthur et al. [11] studied the effect of PREMIER on estimated coronary heart disease (CHD) risk and observed substantial reductions of 12% to 14% in estimated CHD risk, which have important public health benefits. Márquez-Celedonio et al. [12] also found in a study in Mexico that lifestyle modification decreased cardiovascular disease risk in individuals with prehypertension. Prehypertension, defined as blood pressure between 120-139/80-89 mmHg, is a major public health concern [13]. The condition is very prevalent (30% of the adult population), is often associated with other cardiovascular disease risk factors and independently increases the risk of hypertension and subsequent cardiovascular events [13]. The mechanism of elevated risk for cardiovascular events associated with prehypertension is presumed to be the same as that of hypertension [13]. The Seventh Report of the Joint National Committee on Hypertension recommended only lifestyle changes for most prehypertensive patients [14].

To have a significant public health impact, benefits of proven lifestyle interventions have to be translated into community settings [15,16]. Evidence from a varied community, including church-based settings have shown that lifestyle interventions can be effective in lowering diabetes and hypertension risk. Ackerman et al. [17] found positive results in an implementation, evaluation of the delivery of a group-based Diabetes Prevention Programme (DPP) lifestyle intervention in a community setting. Boltri et al. [18] describes the successful translation of the National Institutes of Health Diabetes Prevention Programme (DPP) in African American churches. A 6-session modified DPP was associated with decreased fasting glucose and weight similar to the 16-session programme. Simmons et al. [19,20] was able to reduce risk factors for diabetes among Western Samoans in New Zealand based on a church-based programme. Likewise, Oexmann et al. [21] describes a successful church-based approach to lifestyle change in cardiovascular disease risk in African Americans. The church is an important community intervention setting for health promotion in South Africa [22-24]. There is a lack of evidence that such a community (church-based) lifestyle intervention to lower diabetes and hypertension risk is effective in an African setting. Therefore, a cluster randomized controlled implementation trial to control high normal blood pressure and/or high normal blood glucose in church members in South Africa is proposed. This proposed study will also identify culturally and regionally specific approaches (and constraints) to the translation of the key elements of the lifestyle arm of the Diabetes and Hypertension Prevention Programme, including a determination of the feasibility of and best models for the delivery of physical activity and dietary interventions in a community setting (churches).

### Aim of the study

The aim of this study is to study the efficacy of a community (church) -based lifestyle intervention programme to control high normal blood pressure and/or high normal blood glucose in church members in a randomized controlled trial in Gauteng, South Africa.

### Objectives of the study

1. To measure non-communicable disease profile, including hypertension and diabetes, health behaviours, weight management and psychological distress of church members;

2. To measure the reduction of blood glucose and blood pressure levels after the intervention;

3. To prevent the development of impaired glucose tolerance and hypertension;

4. To compare health behaviours, weight management and psychological distress, blood glucose and blood pressure levels between the intervention and control groups, and within group during 6, 12, 24 and 36 months during and post intervention.

## Methods/Design

### Study design

**Figure 1:**
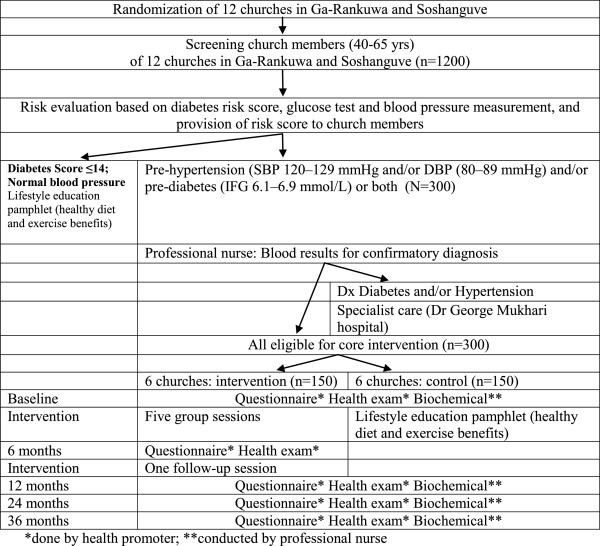
Study design.

The study is a cluster randomized controlled evaluation of a group-based programme, implemented and reported in accordance with the requirements of the CONSORT statement [25] and its extension to cluster randomized trials [26]. From community settings (churches) in Ga-Rankuwa and Soshanguve, 12 churches will be randomly selected for inclusion in the study; 6 churches will be intervention and 6 churches will be control sites (see Figure 1).

#### ***Study hypotheses***

• Individuals receiving lifestyle intervention will have a greater reduction in systolic blood pressure, compared with the individuals receiving a health education leaflet only (control group).

• Individuals receiving lifestyle intervention will have a greater reduction in blood glucose levels, compared with the individuals receiving a health education leaflet only (control group).

### Principles for recruitment

#### ***Inclusion criteria***

##### 

**Churches** Churches having a church building and having at least 100 church members in the locations of Ga-Rankuwa and Soshanguve will be included in the study.

##### 

**Church members** Males and females, aged from 40–65 years, who visit the church, and having been diagnosed with pre-diabetes and/or prehypertension will be included in the study.

#### ***Exclusion criteria***

##### 

**Church members** Church members under the age of 40 and above 65 years will not be included. In addition, all individuals with previous diagnosis of heart diseases, previous diagnosis of hypertension and/or type 2 diabetes, unstable pulmonary disease, history of alcoholism, drug abuse, or other psychiatric problems that are likely to limit compliance to the study, orthopaedic or rheumatologic disease (i.e. severe rheumatoid arthritis), already enrolled in a clinical research trial, and having kidney disease will be excluded.

#### ***Randomisation***

A list of the names of churches in each village/ward will be obtained. The church sites will be randomized into intervention and control sites to prevent contamination. Randomisation will be conducted using a secure remote randomization service. We believe that there will not be any contamination between the experimental and control groups since religious leaders and small group church members will stay nearby their church area. The control and intervention churches will not be matched. There are about 452 Christian churches in Soshanguve and Ga-Rankuwa [27]. In more than half of the churches (52%) church members meet in a church building, more than 70% have 100 or more church members and more than 80% of the church members attend church at least once a week [27].

#### ***Blinding***

Church leaders will be informed that their assignment to conditions is random, and that different churches will receive different types of interventions. Random assignment will occur prior to the baseline assessment to assure that recruitment and assessment staff (research assistant nurses) remains blind to condition assignments.

#### ***Procedure***

After randomization, baseline measurements will occur. Recruitment of the programme participants will take place in 12 churches by specially trained research assistant nurses. Church members will be screened for prehypertension and prediabetes by the research assistant nurses.

### Step 0: Pre-screening

All church members will pass brief pre-screening questions to meet the *Inclusion Criteria*: 1) age from 40–65 years, 2) willingness to participate in the screening programme.

### Step 1: Screening for prehypertension and pre-diabetes (at churches)

The participants will be recruited on Saturday and Sunday after the worship activities. Research assistant nurses will be trained for 1 week on risk factors, data collection and research process. They will screen church members in the church area (by putting up a tent with 4 sides wall), using a diabetes risk screen and blood pressure assessment after informed consent has been taken for study participation. Prehypertension will be defined as blood pressure between 120-139/80-89 mmHg [28]. Blood pressure (BP) is measured with a validated automated digital BP monitor (BpTRU) based on South African guidelines [29] three times at two different visits each, a week apart. Prediabetes is initially assessed with the Diabetes Risk Score Tool. The risk status is determined by a risk factor questionnaire developed on the basis of epidemiological studies [30,31]. A score of 15 or more will be used as a recruitment criterion. High risk equals have an estimated one in three chance of getting the disease during the following 10 years. In the absence of a diabetes risk screen developed in an African population, the Finnish diabetes risk score (FINDRISC) questionnaire will be used [30,31]. In a recent systematic review of risk scores based on self-reported or available clinical data to detect undiagnosed type 2 diabetes found that “An important, but tentative conclusion (given the small number of studies) is that there is no reason to believe that risk scores in low and middle income settings perform differently compared to high income settings” [32], p. 382.

After completion of the face-to-face administered diabetes risk screen, the research assistant nurse will calculate the risk score and provide the results to the client. Likewise, church member’s blood pressure will be measured by a research assistant nurse and the results provided to the client. Clients who score less than 15 on the diabetes risk screen are provided with a lifestyle education pamphlet in English and SeTswana explaining the benefits of a healthy diet and exercise. Likewise, clients with a normal blood pressure are provided with a lifestyle education pamphlet explaining the benefits of a healthy diet and exercise.

Clients who score 15 or more on the diabetes risk screen have elevated diabetes risk. Likewise, clients with a blood pressure between 120-139/80-89 mmHg are at elevated risk for hypertension. The research assistant nurse will take informed consent for participation in the study (Step II). The appointment will be made for the following week at the churches and fasting procedures are explained.

### Step II: Confirmatory testing

A professional nurse will measure blood pressure (three times) and collect fasting venous blood samples from the participants for fasting plasma glucose (FPG) and Lipid profile test. An appointment will be made to receive their results after one week and to recruit into the trial. The Eligibility for Step III are: had high normal blood pressure determined as a seating resting systolic blood pressure between 120 and 139 mmHg and/or a diastolic blood pressure of 80–89 mmHg, and/or a fasting glucose level between 6.1 and 6.9 mmol/l and agreed to participate over a 36-month period (2^nd^ inform consent (for intervention)). Patients with type 2 diabetes and/or hypertension exit the study and are referred for medical management (see Figure 1).

The study intervention will be conducted in church buildings led by trained health promoters (details in the intervention section).

### Consent

Consent to participate will be obtained in a 3-stage process. Research assistant nurses will initially ask for informed consent to conduct a health screen (diabetes risk screen and blood pressure test) and collect basic information and check eligibility to take part. No identifiable information will be collected at this stage. Patients who score positive on the diabetes risk screen and/or have high normal blood pressure, will have the study explained to them verbally and in writing (using the patient information sheet). Informed consent will now be obtained for stage 2 of the study for confirmatory testing of pre-diabetes and/or pre-hypertension. At this second stage, which will include permission to give the contact details to the research assistant nurses staff, and participate in the experimental or control condition and follow ups.

### Interventions

#### ***Lifestyle education leaflet***

All the church members randomized and allocated to the control group will complete the baseline and follow-up measures, receive standard care and a lifestyle education pamphlet on healthy diet and exercise benefits.

#### ***Lifestyle intervention***

In the 6 lifestyle intervention study churches two lay health promoters per church will be trained for two weeks with a standardized training programme, training manuals, and practical exercises. In each church the lay health promoter(s) is expected to start and run 4–5 support groups (6–8 members per group) over the project period. The intervention model used is based on a Finnish diabetes prevention implementation study [33,34] and includes components of the PREMIER study [11,35]. Briefly, the programme consists of six two-hour group sessions facilitated by trained lay health promoters [34]. They will receive two weeks of training with a standardized training programme, training manuals, and practical exercises. The first five sessions will extend over 8 weeks, with 2-week intervals in between sessions. The last session will take place at 6–8 months [34]. The sessions will be facilitated by specially trained lay health promoters, dieticians and physiotherapists. The group counselling sessions provide individuals with dietary and physical activity guidance based on the South African food-based dietary guidelines for adults (South African food-based dietary guidelines) [36] and South African physical activity recommendations [37]. The Health Action Process Approach (HAPA) model [38] and self-regulation theory [39] are used to set individual goals and motivate participants to progress from intention to actual behaviour change to accomplish the diet and physical activity goals of the programme [34]. Programme goals include seven specific goals related to diet and physical activity [35,40]. The goals are: (1) no more than 30 per cent of total energy from fat; (2) no more than 10 per cent of total energy from saturated fats; (3) at least 15 g/1000 kcal fibre; (4) at least 30 min/day moderate intensity physical activity; (5) at least 5 per cent reduction of present body weight, (6) limit daily sodium intake to ≤2400 mg per day and (7) the goal for alcohol intake was ≤2 drinks/day for men and ≤ 1 drink/day for women [35,40]. The lifestyle intervention content areas [11,33-35] are summarized in Table 1.

**Table 1:** Lifestyle intervention contents

**Session**	**Major intervention content**
1	-Lifestyle influence health, diabetes, hypertension, risk factors and development, effects, prevention
	-Goals, planning, homework and other exercises, including sensible alcohol use
	-Homework assignments: monitoring own behaviour with food diary (including identifying sodium content of foods) and physical activity schedule
2	-Returning of food diaries
	-Comparison of own habits with the diet and physical activity goals sufficient for prevention, Role modelling, analysis and re-attribution
	-Homework assignments: preparation for goal setting, monitoring physical activity and eating habits
3	-Feedback from the physical activity schedule
	-Goal planning
	-Homework assignments: feedback and re-enforcement; monitoring physical activity and eating habits
4	-Feedback based on findings from food diaries
	-Education on how to eat healthy?
	-Goal planning, Goal setting
	-Homework assignments: positive feedback in getting social support; monitoring physical activity and eating habits
5	-Evaluating and refining the goals
	-Routines, changed; intermediate goals
	-Exercise: how to overcome barriers, how to use resources in maintaining the behaviour changes
	-Homework assignments: monitoring physical activity and eating habits
6	-Evaluating the goals
	-Routines, changed; analysis and re-attribution of success and failure
	-Future goals and evaluation

#### Assessment and intervention quality assurance

Assessment and intervention quality assurance procedures ensure that project activities are standardized across the churches and across the cohorts and that assessment and intervention process data are collected accurately. To achieve this, standardized protocols, procedures, and training manuals are prepared, and the staff is systematically trained in their use. In addition, the performance of the assessors and programme implementers is routinely monitored by the research coordinators. This will require the development of mechanisms for determining that adequate levels of competence have been attained by those who have received training and that their continuing performance does not fall below these levels. A random sample of group session interactions will be tape-recorded and analysed for that purpose. Feedback will be provided to the field work staff and protocol deviations or other problems addressed and corrected in a timely manner.

### Study measures

The primary outcomes and measures include 1) the reduction in systolic blood pressure after the intervention and 2) the reduction in blood glucose levels after the intervention.

The secondary outcomes and measures include 1) anthropometric measures: body weight, height (for BMI) and waist-to-hip-ratio; body composition 2) lipid levels: total cholesterol, LDL- and HDL-cholesterol, triglycerides; 3) behavioural measures (diet, physical activity, tobacco, alcohol, weight and stress); and 4) blood lipid profiles. Measurements will occur at baseline, 6-months, 12-months and 36-months post intervention (Figure 1). The primary and secondary outcome measures are described as questionnaires, anthropometric and blood pressure measurements and lab tests (see Tables 2 and 3).

**Table 2:** Measures

**Q1-Q5 = Questionnaires: components of the questionnaire include:**	
• Socio-demographic background, Non-communicable diseases, Tobacco use, Physical activity, Food frequency questionnaire, Alcohol use, Dietary behaviour, Dietary practices, Weight management and Perceived body image, and Psychological distress	
• Type II Diabetes Risk Screening	
**A1-A5 = Anthropometric and blood pressure measurements**	
• Weight will be measured on a digital scale to the nearest 0.1 kg in light clothes and without shoes	
• Height will be measured without shoes to the nearest centimeter	
• Waist circumference will be measured (to the nearest 0.1 cm)	
• twice midway between the lowest rib and iliac crest and the mean value will be used	
• Hip circumference will be measured twice at the largest part of the hip, the mean value will be used	
• Body Composition: will be measured using Body Stats machine	
• Blood pressure (BP) is measured with a validated automated digital BP monitor (BpTRU) based on South African guidelines [29]	
**L1-L4 = Lab tests**	
• Fasting venous samples are drawn at the baseline, at three months, 12, 24 and 36 months. All participants are required to fast overnight prior to each clinical test for a minimum of eight hours	
• The following methods are used for blood chemistry: (1) serum total cholesterol: (2) HDL-LDL cholesterol, (3) triglycerides	
• The plasma glucose and lipid profile determinations are carried out using a uniform glucose oxidase-peroxidase and a cholesterol oxidase-phenol aminophenazone (CHOD-PAP) method, respectively	

**Table 3:** Study time line and data collection

**Q1**		**Q2**		**Q3**	**Q4**	**Q5**
L1				L2	L3	L4
A1		A2		A3	A4	A5
	I1-I4	I5	I6			
0 months	1 month	6 ms#	8 ms	12 ms	24 ms	36 ms

#in intervention group only.

### I1-I6 = Intervention sessions (see above)

The fidelity of programme delivery is measured with a facilitator questionnaire after each completed intervention period.

### Sample size calculation

As reviewed by Petrella et al. [41], data from previous intervention studies found a change in systolic blood pressure after the intervention between 5.32 and 5.92 mmHg providing a 90% power [34], while a reduction of blood glucose levels of 0.1 to 0.3 mmol. are noted as important in terms of the risk for the development of type 2 diabetes [35]. The Trial Protocol Tool for RCTs software (2004) was used to calculate sample size at power 90%, average cluster size 20, A median intracluster correlation (ICC) of 0.005 (average Standard Deviation 0.3), as suggested by Adams and Gulliford [42], was used assuming a 80% success rate in achieving suitable recordings in two test sites the minimum unadjusted sample size is 96 and number of clusters per group is 6. From previous similar researches, the dropout rate of approximately 20%, hence recruit 120 subjects; for preventing of loss of two follow up, we increase to 150 subjects in 6 clusters per treatment arm.

### Intervention evaluation and statistical analysis

The RE-AIM (Reach, Efficacy/Effectiveness, Adoption, Implementation, and Maintenance) evaluation framework for complex implementation trials will be utilized to analyze the reach, effectiveness, adoption, and implementation of the intervention [18]. Nutritional intake is analysed by a registered dietitian using Nutrica software (Norfoods, 2000). Evaluation will be conducted in terms of attainment of the diet and physical activity goals; changes in the most significant automated biological markers (blood glucose levels, cholesterol, blood pressure, weight and waist circumference); and changes in the determinants of behaviour.

The statistical analysis will include differences among the subjects’ characteristics in clinical, behavioural, and physiological outcome measures using analysis of variance (ANOVA). Interactions and/or a specific age, gender, or clinical variable differences will be examined using Tukey’s post hoc analyses. If a group difference between a potentially confounding variable is observed at limit differences in a key outcome, analysis of covariance (ANCOVA) will be performed with the potentially confounding variable serving as the covariant. Relations of interest will be initially identified by univariate correlation analysis. Independent correlations among the dependent variables will be determined using partial correlation analyses and/or conventional multivariate step-wise regression analysis. In all cases, probability levels will be p < 0.05.

#### ***Ethical and research governance approval***

We have received ethical approval from the University of Limpopo MEDUNSA Research and Ethics Committee (Project number: MREC/H236/2012:IR).

#### ***Project timescales***

The study will run for a period of 46 months beginning in January 2014 to October 2017.

## Discussion

This integrative lifestyle intervention trial for both the reduction of diabetes and hypertension risk offers the unique opportunity to evaluate the effect of a community (church) -based intervention delivered by trained lay health promoters targeting a high risk population. The integration of a lifestyle intervention for both the reduction of diabetes and hypertension risk and evaluating the effectiveness of this intervention in a community-based setting is both innovative and can facilitate research translation into primary health care practice. Moreover, this intervention utilizes community health workers to enhance lifestyle modification for the reduction of both diabetes and hypertension risk through group sessions, which can be easily adapted in real world settings. This study will present a new analysis of the effectiveness a community (church)-based lifestyle intervention programme to control high normal blood pressure and/or high normal blood glucose in a low and middle income setting. If data confirm our hypothesis, this may help policy makers to increase resources in prevention rather than treatment. It is important to consider that the study data will be collected from a real life community setting.

## Abbreviations

BP: Blood pressure; CHD: Coronary heart disease; DASH: Dietary approaches to stop hypertension; DPP: Diabetes prevention programme; FBS: Fasten blood sugar; IFG: Impaired fasting glucose; IGT: Impaired glucose tolerance.

## Competing interests

The authors declare that they have no competing interests.

## Authors’ contributions

SP and KP were the main contributors to the conceptualization of the study and also contributed significantly to the first draft of the paper. All authors contributed to the subsequent drafts and finalization. All authors read and approved the final manuscript.

## Pre-publication history

The pre-publication history for this paper can be accessed here:

http://www.biomedcentral.com/1471-2458/14/568/prepub
